# Outer Membrane Vesicles From Probiotic and Commensal *Escherichia coli* Activate NOD1-Mediated Immune Responses in Intestinal Epithelial Cells

**DOI:** 10.3389/fmicb.2018.00498

**Published:** 2018-03-20

**Authors:** María-Alexandra Cañas, María-José Fábrega, Rosa Giménez, Josefa Badia, Laura Baldomà

**Affiliations:** ^1^Secció de Bioquímica i Biología Molecular, Departament de Bioquímica i Fisiologia, Facultat de Farmàcia i Ciències de l’Alimentació, Universitat de Barcelona, Barcelona, Spain; ^2^Institut de Biomedicina de la Universitat de Barcelona – Institut Recerca Sant Joan de Deu, Barcelona, Spain

**Keywords:** gut microbiota, *Escherichia coli* Nissle 1917, NF-κB activation, bacterial extracellular vesicles, NOD1

## Abstract

Gut microbiota plays a critical role in maintaining human intestinal homeostasis and host health. Bacterial extracellular vesicles are key players in bacteria–host communication, as they allow delivery of effector molecules into the host cells. Outer membrane vesicles (OMVs) released by Gram-negative bacteria carry many ligands of pattern recognition receptors that are key components of innate immunity. NOD1 and NOD2 cytosolic receptors specifically recognize peptidoglycans present within the bacterial cell wall. These intracellular immune receptors are essential in host defense against bacterial infections and in the regulation of inflammatory responses. Recent contributions show that NODs are also fundamental to maintain intestinal homeostasis and microbiota balance. Peptidoglycan from non-invasive pathogens is delivered to cytosolic NODs through OMVs, which are internalized via endocytosis. Whether this pathway could be used by microbiota to activate NOD receptors remains unexplored. Here, we report that OMVs isolated from the probiotic *Escherichia coli* Nissle 1917 and the commensal ECOR12 activate NOD1 signaling pathways in intestinal epithelial cells. NOD1 silencing and RIP2 inhibition significantly abolished OMV-mediated activation of NF-κB and subsequent IL-6 and IL-8 expression. Confocal fluorescence microscopy analysis confirmed that endocytosed OMVs colocalize with NOD1, trigger the formation of NOD1 aggregates, and promote NOD1 association with early endosomes. This study shows for the first time the activation of NOD1-signaling pathways by extracellular vesicles released by gut microbiota.

## Introduction

There is strong scientific evidence that the gut microbiota has a central role in the well-being of the host. Indeed, this microbial community is a key orchestrator of the immune system, contributing to maintenance of tolerogenic immune responses (Maynard et al., 2012). Communication between microbiota and intestinal epithelial cells is essential to preserve proper microbiota balance and intestinal homeostasis. Under healthy conditions, this inter-kingdom communication is mediated through secreted bacterial compounds that, unlike whole bacteria, can diffuse through the intestinal mucus layer and interact with epithelial cells (Sánchez et al., 2010; Hevia et al., 2015). Besides soluble proteins, Gram-negative bacteria also release active mediators into the intestinal lumen through OMVs (Ahmadi-Badi et al., 2017). Most studies have been focused on pathogen released OMVs and have proven their role in virulence (Yoon et al., 2011; Pollak et al., 2012; Bielaszewska et al., 2013; Jäger et al., 2015; Schwechheimer and Kuehn, 2015; Jung et al., 2016).

In contrast, vesicles released by commensal and probiotic bacteria have been associated with beneficial effects for the host. Therefore, it has been suggested that microbiota vesicles may act as key players to maintain intestinal homeostasis (Kaparakis-Liaskos and Ferrero, 2015; Patten et al., 2017). However, there are relatively few reports on this field. Studies performed with prevalent Gram-negative bacteria that reside in the human gut showed that OMVs released by *Bacteroides fragilis* and *Akkermansia muciniphila* promote immunomodulatory effects and prevent gut inflammation in mice models of experimental colitis (Shen et al., 2012; Kang et al., 2013). In this context, we have proved that OMVs from the probiotic EcN and other commensal *E. coli* strains deliver mediators that trigger host immune and defense responses. These vesicles are internalized by intestinal epithelial cells via clathrin-mediated endocytosis and sorted to lysosomes through endocytic compartments (Cañas et al., 2016). OMVs from microbiota *E. coli* strains exhibit immunomodulatory activity on different *in vitro* models of intestinal barrier and in human colonic explants, regulating expression of antimicrobial peptides and inflammatory biomarkers toward an anti-inflammatory profile (Fábrega et al., 2016). Oral administration of OMVs isolated from the probiotic EcN ameliorate inflammation and colitis progression in DSS-treated mice, similarly to the administration of probiotic suspensions (Fábrega et al., 2017). In addition to immune modulation, EcN OMVs reinforce the intestinal barrier and reduce gut permeability by promoting upregulation of tight junction proteins ZO-1 and claudin-14, and downregulation of claudin-2 (Alvarez et al., 2016).

Despite these early findings that indicate a key role of bacterial vesicles in signaling processes at the intestinal mucosa, the specific molecular mechanisms and pathways involved in microbiota OMVs-host crosstalk remain largely unexplored. In this regard, vesicles are loaded with MAMPs including LPS, peptidoglycan, lipoproteins, DNA and RNA, which allow OMVs to interact directly with host cells via PRRs and activate signaling pathways that result in cytokine/chemokine modulation (Kaparakis-Liaskos and Ferrero, 2015; Patten et al., 2017). Besides recognition of extracellular TLRs, commensal bacteria can also signal through cytosolic NLRs, which include two members, NOD1 and NOD2 (Natividad et al., 2012).

NOD1 is constitutively expressed in most cell types and especially by epithelial cells in the intestinal tract, whereas NOD2 is mainly expressed in immune cells of myeloid origin. However, low expression of NOD2 has also been reported in intestinal epithelial cells, where certain stimuli such as pro-inflammatory cytokines or infection with enteroinvasive pathogens can up-regulate its expression (Rosenstiel et al., 2003; Kim et al., 2004). NOD receptors consist of three domains: (i) an N-terminal caspase-activated recruitment domain (CARD) that allows protein–protein interactions, (ii) a central NACHT domain involved in receptor dimerization, and (iii) a C-terminal leucine-rich repeat (LRR) domain that recognizes specific ligands. Both NOD1 and NOD2 are activated by intracellular fragments of bacterial peptidoglycan, thereby acting as intracellular sensors of bacterial infection. NOD1 detects D-glutamyl-meso-diaminopimelic acid (iE-DAP) present in Gram-negative PG, but also in specific groups of Gram-positive bacteria, while NOD2 detects muramyl dipeptide (MDP), which is common to all groups of bacteria. PG interaction elicits NOD oligomerization, which in turn promotes the recruitment of the downstream RIP2, a specific kinase that triggers activation of NF-κB and mitogen-activated protein kinase (MAPK) pathways leading to the expression of inflammatory genes. NOD receptors have essential roles not only in host responses against bacterial infection, but also in the regulation of intestinal inflammatory response to microbiota, and thus in maintaining intestinal homeostasis (reviewed in Caruso et al., 2014; Philpott et al., 2014; Kaparakis-Liaskos, 2015).

In addition to PG entry through cell infection by invasive bacteria, other intracellular PG delivery pathways have been described in non-invasive bacteria. These alternative routes include PG translocation through bacterial secretion systems, uptake by endocytosis or by specific membrane transport systems (PEPT), and delivery via OMVs (reviewed in Philpott et al., 2014; Kaparakis-Liaskos, 2015). Delivery of PG inside the host cell and further activation of NOD signaling cascades by bacterial OMVs have been studied in pathogens, specifically *Helicobacter pylori* (Kaparakis-Liaskos et al., 2010), *Vibrio cholerae* (Chatterjee and Chaudhuri, 2013), and *Aggregatibacter actinomycetemcomitans* (Thay et al., 2014). OMVs from these pathogens induce an inflammatory response that is NOD1-dependent. Studies performed with *H. pylori* OMVs revealed that delivery of endocytosed PG-containing OMVs to cytosolic NOD1 is triggered by recruitment of the cytosolic receptor to early endosomes. This intracellular trafficking was shown to be essential for NOD1-PG interaction and activation of the signaling cascade that leads to nuclear NF-κB translocation (Irving et al., 2014).

Intracellular PG delivery by means of bacterial extracellular vesicles points to a plausible mechanism used by microbiota to activate NOD-mediated host immune responses. The aim of the present study was to evaluate whether OMVs from probiotic and commensal *E. coli* could activate NOD signaling pathways in intestinal epithelial cells. Here, we show that in Caco-2 cells NOD1, but not NOD2, is essential for the immune responses mediated by EcN or ECOR12 OMVs. OMV-mediated activation of NF-κB and the subsequent IL-6 and IL-8 responses are significantly reduced by NOD1 silencing or RIP2 inhibition. Colocalization of endocytosed OMVs with NOD1 was confirmed by confocal fluorescence microscopy. This analysis also provides evidence of NOD aggregation and association with early endosomes in cells stimulated with EcN or ECOR12 OMVs.

## Materials and Methods

### Bacterial Strains and Growth Conditions

The probiotic EcN (serotype O6:K5:H1) was provided by Ardeypharm GmbH, Herdecke, Germany. ECOR12 (O7:H32) is a strain of the *E. coli* reference collection that was isolated from the stool of a healthy human (Ochman and Selander, 1984). Bacterial cells were routinely grown at 37°C in LB with constant rotation (150 rpm). Growth was monitored by measuring the optical density at 600 nm.

### Isolation and Labeling of OMVs

OMVs were isolated from culture supernatants as described previously (Aguilera et al., 2014). Briefly, bacterial strains were grown aerobically in LB and collected by centrifugation at 10,000 × *g* for 20 min at 4°C. The supernatants were filtered through a 0.22 μm-pore-size filter (Merck Millipore) to remove remaining bacteria and concentrated using a 10k Centricon^®^ Plus-70 filter unit (Merck Millipore). Vesicles were pelleted by centrifugation at 150,000 × *g* for 1 h at 4°C in an Optima^TM^ L-90K ultracentrifuge (Beckman Coulter), washed twice, resuspended in PBS and stored at -20°C. Sterility of samples was assessed on LB plates. Protein concentration was measured by the method of Lowry et al. (1951). The reproducibility of OMV preparations was assessed by transmission electron microscopy after negative staining and SDS-PAGE as described previously (Aguilera et al., 2014).

Outer membrane vesicles were fluorescently labeled with BODIPYFL vancomycin (Thermo Fisher Scientific), which selectively reacts with PG. To this end, EcN and ECOR12 OMVs (2 mg/ml) were incubated for 1 h at room temperature in the presence of 4 ng/ml of BODIP^®^YFL vancomycin. Unbound dye was removed by washing two times in PBS. Labeled OMVs were then collected by centrifugation at 100,000 × *g* for 1 h at 4°C.

### Cell Culture and Stimulation Conditions

The human colonic cells lines Caco-2 (ATCC HTB37) and HT-29 (ATCC HTB-38) were from the American Type Culture Collection. Previous studies of our group showed that EcN and ECOR12 OMVs are internalized by both intestinal epithelial cells through clathrin-mediated endocytosis, ruling out cell-dependent differences in the endocytic and intracellular trafficking pathways (Cañas et al., 2016). Epithelial cells were cultured in DMEM High Glucose (Life Technologies) supplemented with 10% (v/v) fetal bovine serum (FBS), 25 mM HEPES, 1% non-essential amino acids, penicillin (100 U/ml) and streptomycin (100 μg/ml) (Gibco BRL, Gaithersburg, MD, United States). Cultures were incubated at 37°C in a 5% CO_2_ atmosphere. Cells were routinely sub-cultured once a week with trypsin-EDTA (0.25%, 0.53 mM) and seeded at a density of 2 × 10^5^ cell/ml.

For gene silencing, Caco-2 intestinal epithelial cells (2 × 10^5^ cell/ml) were seeded in 12 well tissue culture plates and grown until 70–80% of confluence. Then, medium was replaced with antibiotic-free medium, and cells were transfected with specific NOD1 (Santa Cruz Biotechnology sc-37279) or NOD2 (Santa Cruz Biotechnology sc-43973) small interfering RNA (siRNA) following the manufacturer’s instructions. A negative control siRNA (Santa Cruz Biotechnology sc-37007) was used as reference. The transfection and medium reagent used in this study were also obtained from Santa Cruz Biotechnology. After 48 h, the culture medium was replaced with fresh DMEM and NOD1 and NOD2 knockdown Caco-2 cells were stimulated with EcN or ECOR12 OMVs, at dose of 10 μg/ml, for 8 h. To inhibit RIP2, Caco-2 cells were treated with 1 μM of RIP2 tyrosine kinase inhibitor Gefitinib (Invivogen) for 1 h prior the addition of OMVs (10 μg/ml), and then incubated in the presence of the inhibitor for an additional 8 h. As a positive control Caco-2 cells (control, NOD1 silenced, and Gefitinib treated cells), were stimulated for 8 h with 1 μg/ml of M-TRiDAP (MurNAc-L-Ala-gamma-D-Glu-mDAP) (Invivogen), a PG degradation product found mostly in Gram-negative bacteria that is recognized by the intracellular receptor NOD1. In all cases, supernatant and cells were collected for ELISA and quantitative RT-PCR assays, respectively.

### Analysis of NF-κB Activation in Caco-2 Cells

To measure activation of the nuclear transcription factor NF-κB, control Caco-2 cells as well as cells treated with the RIP-2 inhibitor or transfected with NOD1 siRNA were stimulated with 10 μg/ml of EcN and ECOR12 OMVs at different timings (20, 40, 90, 120, 150, and 180 min). Then, cells were washed with 1X PBS and suspended in lysis buffer (50 mM Tris-HCl (pH 8.0), 170 mM NaCl, 0.5% NP_4_O, 50 mM NaF, 25% glycerol, 10 mg/ml proteinase A, 10 mg/ml leupeptine, 1 mM DMSF, 0.1 mM Na_3_, 20 mM β-glycerolphosphate, and 1 mM DTT) for 1 h at 4°C. Cell lysate was clarified by centrifugation at 16,000 × *g* for 30 min at 4°C. Protein concentration was measured by the Bradford Protein Assay (Bio-Rad). NF-κB activation was evaluated through IkBα degradation by Western blot. Samples (100 μg protein) were mixed with SDS-PAGE sample loading buffer, boiled for 5 min at 95°C and electrophoresed on 10% SDS-PAGE gel. Proteins were transferred to a Hybond-P polyvinylidene difluoride membrane using a Bio-Rad Mini Transblot apparatus. The membrane was blocked in PBS-0.05% Tween-20 and 5% skimmed milk (blocking solution) for 1 h at room temperature, and incubated with specific antibodies against IκBα (Santa Cruz Biotechnology), 1:500 dilution in blocking solution overnight at 4°C, followed by incubation with peroxidase-conjugated anti-mouse IgG antibody (1:7000) for 1 h at room temperature. The antibody-protein complex was visualized using the ECL Plus Western blotting detection system (GeHealthcare). Detection of the housekeeping NEK9 protein was used as an internal loading and transfer control.

### Confocal Fluorescence Microscopy

HT-29 cells were grown in 8-well chamber slider (Ibidi) until approximately 80% confluence and incubated with EcN and ECOR12 OMVs (10 μg/well) at 37°C. When indicated cells were incubated with BODIPYL-FL labeled OMVs. After stimulation, cells were washed three times in PBS. Nuclei were labeled with DAPI (0.125 μg/ml, Sigma-Aldrich). Then, samples were fixed with 3% paraformaldehyde for 30 min, permeabilized with 0.05% saponin (Sigma-Aldrich) and blocked with PBS containing 1% bovine serum albumin for microscopy analysis. Unlabeled OMVs were visualized using anti-*E. coli* LPS mouse monoclonal antibody (Abcam^®^) followed by Alexa Fluor 488 conjugated goat anti-mouse IgG (Molecular Probes). NOD1 receptor was detected using anti-NOD1 policlonal antibody (Thermofisher) and Alexa Fluor 633 conjugated goat anti-rabbit IgG (Molecular Probes). Endosomes were labeled with mouse polyclonal antibody against EEA1 (Santa Cruz Biotechnology) and Alexa Fluor 488-conjugated goat anti-rabbit IgG (Molecular Probes).

Immunofluorescence labeling of cells was analyzed by confocal microscopy using a Leica TCS SP2 laser scanning confocal spectral microscope with a 63x 1.32NA oil immersion objective and an image resolution of 0.232 × 0.232 × 0.488 μm/voxel (*x, y, z*, respectively). Images were captured with a Nikon color camera (8 bit). Fluorescence was recorded at 405 nm (blue; DAPI), 488 nm (green; BODIPYL-FL, Alexa Fluor 488), 546 nm (red; WGA), and 633 nm (far-red; Alexa Fluor 633). Images were analyzed using Fiji image processing package (Schindelin et al., 2012). Colocalization was analyzed by calculating the overlap coefficient (r), which values range from 0 to 1. For the calculation, data were obtained from four confocal stacks (x-y) using the JaCoP plugin. The total number of cells analyzed was between 60 and 70. Data were presented as mean ± standard error (SEM) from three independent biological experiments.

### Cytokine Quantification

After stimulation, culture supernatants were collected and centrifuged at 16,000 × *g* for 20 min at 4°C, and stored at -80°C until assay. IL-8 and IL-6 secreted levels were quantified by enzyme-linked immunosorbent assay (ELISA) sets (BD Biosciences) according to manufacturer’s instructions. The results were expressed as pg/ml.

### RNA Isolation and Quantitative Reverse Transcription PCR (RT-qPCR)

Total RNA was isolated from Caco-2 cells using the Illustra RNAspin Mini kit (GE Healthcare) according to the manufacturer’s protocol. Purity and RNA concentration were assessed by the absorbance ratio at 260 and 280 nm in a NanoDrop^®^ spectrophotometer. RNA integrity was evaluated by visualization of 28S and 18S rRNAs after 1% agarose/formaldehyde gel electrophoresis.

RNA (1 μg) was reverse transcribed using the High Capacity cDNA Reverse Transcription kit (Applied Biosystems) in a final volume of 20 μl. RT-qPCR reactions were performed in a StepOne Plus PCR cycler (Applied Biosystems) by using SYBR Green PCR Master Mix (Applied Biosystems) and specific primers for human IL-6, IL-8, NOD-1, and NOD-2 (**Table 1**). A control reaction was performed in the absence of RNA. The 2^-ΔΔCt^ method was used to normalize expression results. The expression level of the housekeeping β-actin gene was used as a reference to normalize the expression values of the genes under study.

**Table 1:** Primer sequences used for RT-qPCR.

Gene	Primer sequence	Reference
NOD1	5′-ACGATGAAGTGGCAGAGAGTT-3′	Boonyanugomol et al., 2013
	5′-GGCAGTCCCCTTAGCTGTGA-3′	
NOD2	5′-GAAGTACATCCGCACCGAG-3′	Sabbah et al., 2009
	5′-GACACCATCCATGAGAAGACAG-3′	
IL-8	5′-CTGATTTCTGCAGCTCTGTG-3′	Martirosyan et al., 2013
	5′-GGGTGGAAAGGTTTGGAGTATG-3′	
IL-6	5′-AGCCACTCACCTCTTCAGAAC-3′	Jiang et al., 2017
	5′-GCCTCTTTGCTGCTTTCACAC-3′	
β-Actin	5′-GCTCTGGCTCCTAGCACCAT-3′	Takahashi et al., 2011
	5′-GCCACCGATCCACACAGAGT-3′	


### Statistical Analyses

Statistical analysis was performed using SPSS (version 20.0, Chicago, IL, United States) software package. Results are presented as the mean ± standard error (SEM) of three independent biological experiments, assayed in triplicate. Differences between two groups were assessed using one-way ANOVA followed by Tukey’s test. The *p-*value < 0.05 was considered statistically significant.

## Results

### EcN and ECOR12 OMVs Activate the Innate Inflammatory Response in an NOD1 Dependent Manner in Intestinal Epithelial Cells

In non-invasive pathogens, internalized OMVs allow intracellular delivery of PG to activate NOD receptors upon their recruitment to early endosomes (Irving et al., 2014). Whether microbiota could activate these intracellular receptors through extracellular vesicles remains unknown. Thus, we sought to analyze whether OMVs released by the commensal ECOR12 and the probiotic EcN can trigger the NOD inflammatory response in intestinal epithelial cells.

**FIGURE 1:**
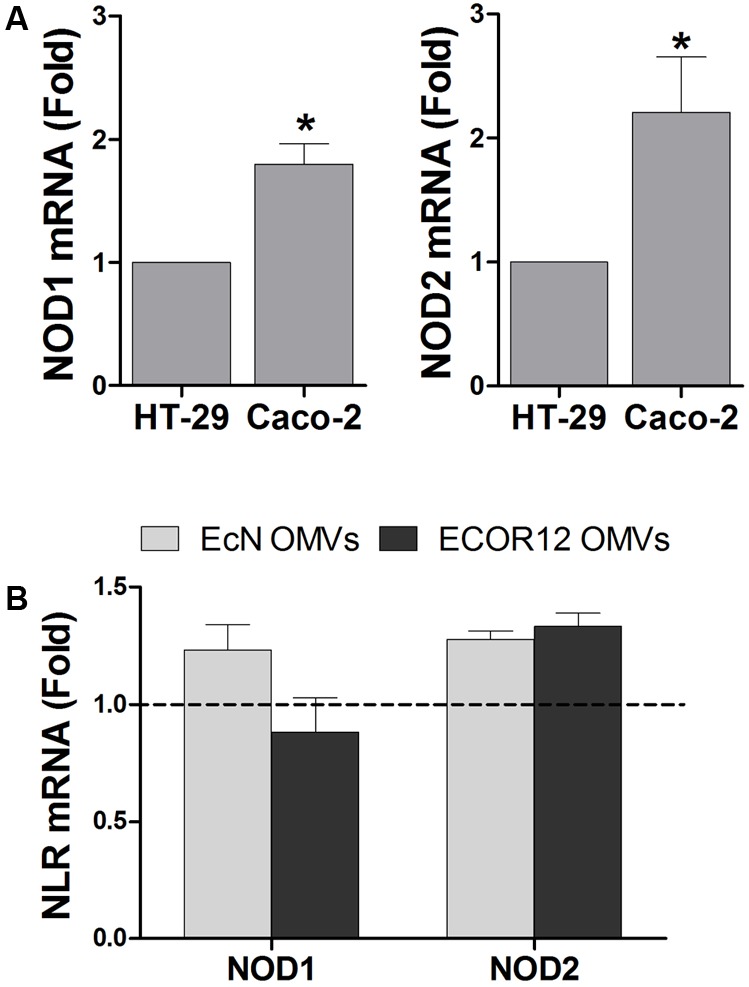
Expression analysis of NOD1 and NOD2 in intestinal epithelial cells. Relative mRNA levels of NOD1 and NOD2 were measured by RT-qPCR, using β-actin as the reference gene. **(A)** Relative mRNA levels of NOD1 and NOD2 in HT-29 and Caco-2 cells. Data correspond to the mean ± SEM of three independent biological experiments and are presented as relative expression in Caco-2 cells in comparison with mRNA levels in HT-29, which were set at 1 (^∗^*p* < 0.010). **(B)** Relative mRNA levels of NOD1 and NOD2 in Caco-2 cells after 8 h-stimulation with OMVs isolated from EcN or ECOR12. Data correspond to the mean ± SEM of three independent biological experiments and are presented as fold-change compared to untreated control cells, whose normalized values were set as 1 and indicated by a dashed line (^∗^*p* ≤ 0.05).

First, we analyzed by RT-qPCR the expression of NOD1 and NOD2 in two intestinal epithelial cells lines, HT-29 and Caco-2. Both intracellular receptors were constitutively expressed in the two cell lines, but their relative expression level was higher in Caco-2 cells (**Figure 1A**). It must be stressed that NOD2 expression was very low. The threshold cycle (Ct) values for NOD2 detection were around 10 times higher than those for NOD1. These results are in accordance with the expression profile reported for these receptors in several epithelial cell lines evaluated by semi-quantitative RT-PCR (Kim et al., 2004).

To approach functional studies, we selected Caco-2 cells as they express higher levels of intracellular NOD receptors than HT-29. In addition, we took advantage of the negligible expression of TLR4 and its co-receptor MD-2 in this cell line (Abreu et al., 2001; Kim et al., 2004). This avoids interference by the other main inflammatory pathways activated by vesicle components other than PG, such as LPS. NOD1 is constitutively expressed in intestinal epithelial cells, while NOD2 is upregulated by treatment with specific pro-inflammatory cytokines or by infection with invasive bacteria. To test whether NOD2 expression could be upregulated in intestinal epithelial cells by internalized OMVs from microbiota strains, the relative expression of both NOD receptors was assessed in Caco-2 cells challenged with EcN or ECOR12 OMVs (10 μg/ml). The basal NOD1 and NOD2 mRNA levels remained unchanged after 8 h stimulation (**Figure 1B**).

To analyze whether internalized OMVs elicit an inflammatory response through activation of NOD1 and/or NOD2 receptors, we examined OMV-induced responses in Caco-2 cells silenced for NOD1 or NOD2 expression. To this end, Caco-2 cells were transfected with siRNA specific sequences targeting these NOD receptors, or with control scrambled siRNA (Scr). After 2 days, knockdown of NOD1 or NOD2 gene expression in these cells was assessed by qRT-PCR. The levels of NOD1 and NOD2 mRNA were significantly decreased by 50 and 55%, respectively, compared with scrambled transfected cells (Supplementary Figure S1). Then, transfected cells were stimulated for 8 h with OMVs from EcN or ECOR12, and the expression of IL-8 and IL-6 was quantified by RT-qPCR. Both bacterial vesicles induced significant levels of IL-6 and IL-8 mRNA expression in control cells (transfected with Scr siRNA).

**FIGURE 2:**
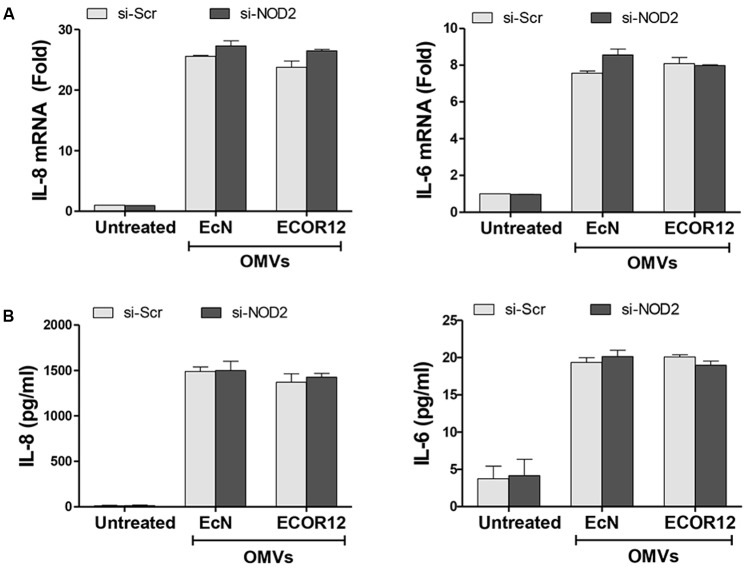
NOD2 is not essential for the OMV-mediated activation of IL-6 and IL-8 expression in Caco-2 cells. Caco-2 cells were transfected with siRNA against NOD2 or with control scrambled siRNA (Scr) as a control. After 48 h, cells were stimulated with OMVs (10 μg/ml) isolated from EcN or ECOR12 strains or left non-stimulated. **(A)** Relative mRNA levels of the pro-inflammatory cytokines IL-6 and IL-8 were measured after 8 h by RT-qPCR, using β-actin as the reference gene. Data are expressed as fold-change compared to non-stimulated Scr-control cells. **(B)** Secreted IL-6 and IL-8 were quantified by ELISA at 8 h post-stimulation. In all panels, data correspond to the mean ± SEM of three independent biological experiments.

**FIGURE 3:**
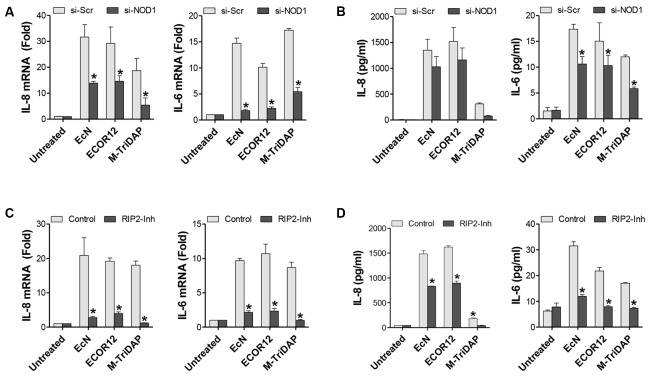
EcN and ECOR12 OMVs activate IL-6 and IL-8 expression in intestinal epithelial cells in a NOD1- and RIP2-dependent manner. Caco-2 cells were stimulated for 8 h with OMVs (10 μg/ml) isolated from EcN or ECOR12 strains or with M-TriDAP (1 μg/ml) as a positive control of NOD1 activation. Control cells were left unstimulated for comparison. **(A,B)** Involvement of NOD1 in the OMV-mediated activation of IL-6 and IL-8 responses was assessed in NOD1-silenced cells. Two days before the stimulation with OMVs or with M-TriDAP, Caco-2 cells were transfected with siRNA against NOD1 or with control scrambled siRNA (Scr). Expression of these cytokines was quantified after 8 h incubation: **(A)** relative mRNA levels of IL-6 and IL-8 were measured by RT-qPCR using β-actin as the reference gene, and **(B)** secreted IL-6 and IL-8 were quantified by ELISA. **(C,D)** Requirement of RIP2 in the OMV-mediated activation of IL-6 and IL-8 responses was assessed in Caco-2 cells treated with the RIP2 inhibitor Gefitinib. Prior to the addition of OMVs or M-TriDAP, cells were pre-treated with Gefitinib (1 μM) for 1 h. Expression of IL-6 and IL-8 was quantified by **(C)** RT-qPCR and **(D)** ELISA as described for NOD1-knockdown cells. In all panels data are presented as mean ± SEM of three independent biological experiments (^∗^*p* ≤ 0.05 *versus* non-stimulated controls). Relative mRNA levels **(A,C)** are expressed as fold-change compared to non-stimulated controls.

NOD2 silencing did not impair OMV-mediated upregulation of either IL-6 or IL-8, as both mRNA and cytokine secreted levels remained similar to those of control Scr-transfected cells (**Figure 2**). In contrast, NOD1 siRNA-transfected cells displayed significantly reduced mRNA levels of IL-6 (sevenfold decrease) and IL-8 (twofold decrease) in response to OMV stimulation (**Figure 3A**). In NOD1-knockdown cells the observed changes in cytokine mRNA expression correlated with the secreted protein levels quantified by ELISA (**Figure 3B**). In response to OMVs, secreted IL-6 and IL-8 levels were lower in NOD1-silenced cells than in Scr-control cells; although for IL-8 the results did not reach statistical significance (**Figure 3B**). Stimulations with Tri-DAP, a cognate activator of the NOD1 signaling pathway, were performed in parallel to assess functional NOD1 activity in these cells. Consistently, IL-6 and IL-8 expression was significantly reduced by NOD1-specific siRNA treatment at both mRNA and protein levels (**Figures 3A,B**). Overall, these results indicate that NOD1, but not NOD2, is involved in the IL-6 and IL-8 responses triggered by internalized EcN or ECOR12 OMVs in intestinal epithelial cells, although NOD1-independent pathways also contribute to IL-8 production.

As RIP2 specifically mediates pro-inflammatory signaling from NOD1 bacterial sensors (Park et al., 2007), we next sought to analyze IL-6 and IL-8 production in response to microbiota OMVs in Caco-2 cells treated with the RIP2 inhibitor Gefitinib. To this end, cells were pre-treated with Gefitinib (1 μM) prior to the addition of EcN or ECOR12 OMVs, and the levels of IL-6 and IL-8 were measured after 8 h-incubation. Inhibition of RIP2 significantly reduced the expression and secretion of both pro-inflammatory cytokines in response to OMVs in Caco-2 cells compared to OMV-stimulated controls (**Figures 3C,D**). These findings support the requirement of the RIP2 kinase in the inflammatory response to internalized microbiota OMVs, and therefore confirm the ability of microbiota OMVs to activate intracellular NOD1 and the downstream signaling pathway.

### EcN and ECOR12 OMVs Activate NF-κB Through the NOD1-RIP2 Pathway

**FIGURE 4:**
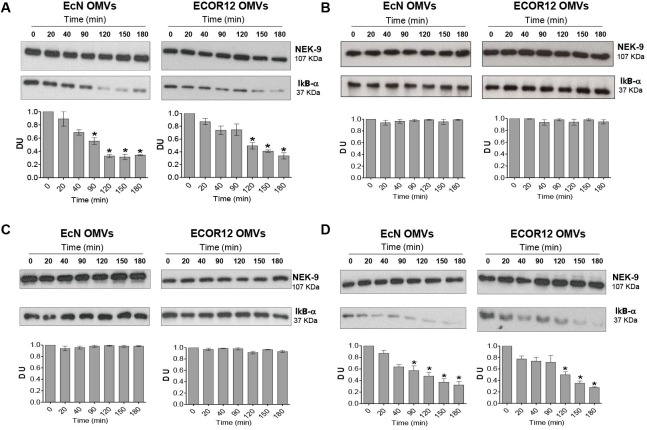
Inhibition of RIP2 impairs OMVs-induced NF-κB activation in Caco-2 cells. Activation of NF-κB was assayed by means of IκBα degradation. **(A)** Caco-2 cells were stimulated with EcN or ECOR12 OMVs (10 μg/ml) for 20, 40, 90, 120, 150 and 180 min, and IκBα levels were assessed by Western blot. For normalization, blots were probed with an anti-NEK-9 antibody. For comparison, parallel stimulations were performed in Caco-2 cells treated with the RIP2 inhibitor Genfitinib (1 μM) **(B)**, transfected with si-NOD1 **(C)**, or transfected with si-NOD2 **(D)**. Densitometric quantification of IκBα was performed with samples from three independent experiments. Representative immunoblots were shown. Normalized values from untreated control cells (time 0 min) were set as 1. Significance against untreated control cells (^∗^*p* ≤ 0.05).

Activation of RIP2 results in the phosphorylation and subsequent degradation of IκBα, which in turn allows activation and translocation of the NF-κB complex into the nucleus, where it induces the transcription of target pro-inflammatory genes (Chin et al., 2002). To confirm activation of NF-κB in cells challenged with OMVs, levels of IκBα were assessed by Western blot at different post-treatment times. Stimulation of Caco-2 cells with EcN or ECOR12 OMVs caused significant degradation of IκBα. The time kinetics of IκBα degradation was somewhat slower for ECOR12 OMVs (**Figure 4A**). Degradation of IκBα was not observed when cells were either transfected with NOD1 siRNA or treated with the RIP2 inhibitor Gefitinib (**Figures 4B,C**). In NOD2-silenced cells, OMV-mediated IκBα degradation followed the same pattern as control Caco-2 cells (**Figure 4D**). These data confirm that OMVs released by *E. coli* microbiota strains activate the NF-κB pathway in an NOD1-dependent manner.

### EcN and ECOR12 OMVs Associate With NOD1 and Trigger Its Aggregation

**FIGURE 5:**
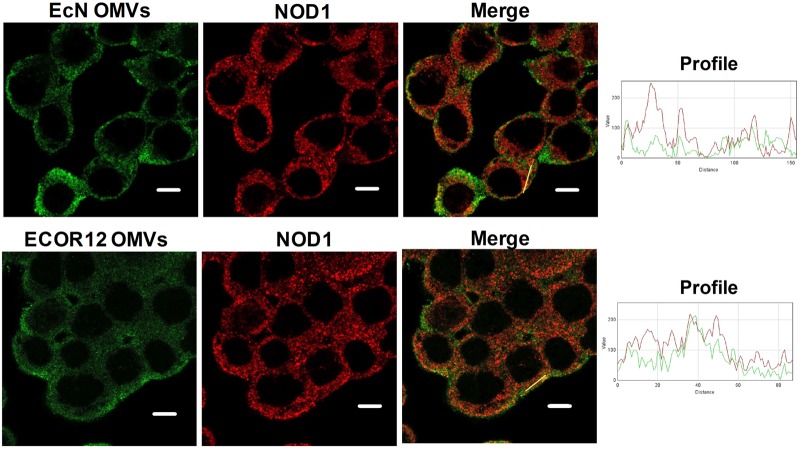
NOD1 colocalizes with EcN and ECOR12 OMVs. HT-29 cells were incubated with EcN or ECOR12 OMVs (10 μg) for 1 h and analyzed using laser scanning confocal spectral microscope. NOD1 was stained using anti-NOD1 polyclonal antibody and Alexa Fluor 633-conjugated goat anti-rabbit IgG (red). OMVs were stained using anti-*Escherichia coli* LPS mouse monoclonal antibody followed by Alexa Fluor 488-conjugated goat anti-mouse IgG (green). Images are representative of three independent biological experiments. Colocalization of the red and green signals was confirmed by histogram analysis of the fluorescence intensities along the yellow lines. Analysis was performed by laser scanning confocal spectral microscope with 63x oil immersion objective lens, and images were captured with a Nikon color camera (8 bit). Scale bar: 10 μm.

NOD1 detection of PG contained in Gram-negative derived OMVs is required for activation of the downstream signaling pathway. Thus, association of NOD1 with OMVs from EcN and ECOR12 was evaluated by means of confocal fluorescence microscopy. OMVs were visualized by immunostaining with anti-*E. coli* LPS and Alexa Fluor 546-conjugated anti-mouse IgG antibodies. After 1 h post-stimulation, OMVs from both strains colocalized with NOD1 in HT-29 cells (**Figure 5**). The degree of colocalization was assessed from the corresponding overlap coefficients (r), with values of 0.51 ± 0.03 for EcN and 0.48 ± 0.01 for ECOR12 vesicles. Colocalization of internalized vesicles with NOD1 was confirmed in HT-29 cells incubated with EcN OMVs fluorescently labeled with BODIP^®^YFL vancomycin (Supplementary Figure S2).

**FIGURE 6:**
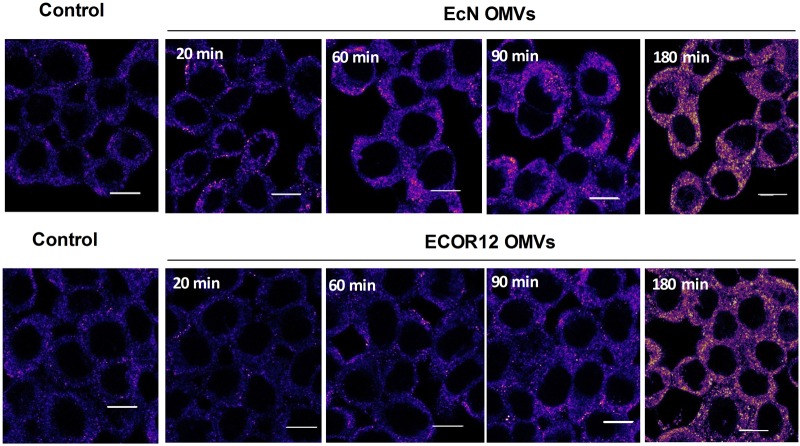
Time-course analysis of NOD1 aggregation pattern in HT-29 cells in response to EcN or ECOR12 OMVs. HT-29 cells were incubated with EcN or ECOR12 OMVs (10 μg) for the indicated times and analyzed using laser scanning confocal spectral microscope. NOD1 was stained using anti-NOD1 polyclonal antibody and Alexa Fluor 633-conjugated goat anti-rabbit IgG. Images are representative of three independent biological experiments and are presented in color-code Fire. Analysis was performed as described for **Figure 5**. Scale bar: 10 μm.

It is well-known that direct binding of PG ligand to NOD1 triggers self-association of the receptor via the NACHT domain. This is a key step for the recruitment of RIP2 to initiate the phosphorylation cascade that results in the assembly of large signaling complexes (Philpott et al., 2014). To address whether microbiota OMVs induce changes in the NOD1 oligomerization state, confocal fluorescence microscopy of NOD1 was performed in HT-29 at different post-incubation times (20, 60, 90, and 180 min). Non-stimulated cells were processed in parallel as a control. These analyses revealed that upon OMVs stimulation, NOD1 formed aggregates in the cell cytoplasm in a time-dependent manner, which may arise by oligomerization upon NOD1 activation (**Figure 6**). Remarkably, NOD1 activation timing profiles differed between OMVs. The pattern of NOD1 aggregation was already apparent at 20 min post-stimulation with EcN OMVs, and gradually increased up to 180 min. However, changes in the NOD1 fluorescence signal pattern were delayed in cells treated with ECOR12 OMVs. At 180 min, both OMVs elicited a similar NOD1 aggregation level. These results are consistent with the different IκBα degradation profile observed in Caco-2 cells in response to these *E. coli* derived OMVs.

### NOD1 Is Recruited to Early Endosomes by EcN and ECOR12 OMVs

**FIGURE 7:**
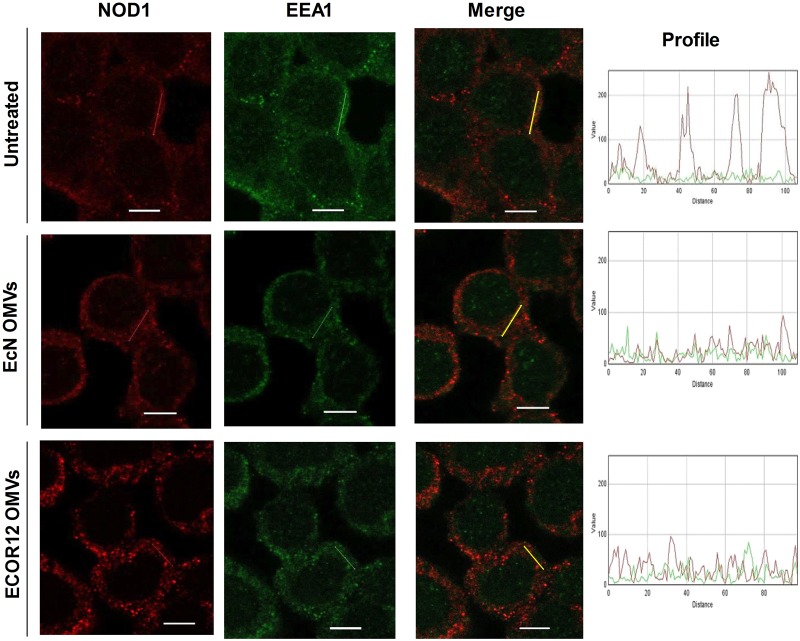
NOD1 colocalizes with EEA1-labeled endosomes in cells stimulated with EcN or ECOR 12 OMVs. HT-29 cells were incubated with EcN or ECOR12 OMVs (10 μg) for 1 h and colocalization of EEA1/NOD1 was analyzed using laser scanning confocal spectral microscope. NOD1 was stained using anti-NOD1 polyclonal antibody and Alexa Fluor 633-conjugated goat anti-rabbit IgG (red). Endosomes were detected with mouse polyclonal antibody against EEA1 and Alexa Fluor 488-conjugated goat anti-rabbit IgG (green). Images are representative of three independent biological experiments. Colocalization of the red and green signals was confirmed by histogram analysis of the fluorescence intensities along the yellow lines. Analysis was performed as described for **Figure 5**. Scale bar: 10 μm.

It has been described that NOD1 detection of OMV-internalized PG takes place at early endosomes (Irving et al., 2014). In this context, we have previously shown that EcN and ECOR12 OMVs are sorted to early endosomes during their intracellular trafficking through clathrin-mediated endocytosis (Cañas et al., 2016). To assess whether NOD1 could be recruited to early endosomes by internalized microbiota OMVs, colocalization of NOD1 with the endosome-associated EEA1 protein was analyzed by confocal fluorescence microscopy in HT-29 cells at 1 h post-stimulation. Non-treated control cells were analyzed in parallel for comparison. Colocalization of NOD1 with the specific endosome marker was only observed in OMVs stimulated cells (**Figure 7**). No yellow spots were apparent in the merged images collected from control cells. In stimulated cells, the presence of OMVs in early endosomes was confirmed by their colocalization with EEA1 (not shown). Quantitative analysis revealed similar overlapping coefficient values for colocalizations of EEA1/NOD1 (*r* = 0.38 ± 0.03 in EcN OMV-treated cells and *r* = 0.34 ± 0.01 in ECOR12 OMV-treated cells) and EEA1/LPS-OMVs (*r* = 0.44 ± 0.08 for EcN OMVs and *r* = 0.39 ± 0.04 for ECOR12 OMVs). These results indicate that NOD1 detection of PG internalized via microbiota OMVs also depends on its recruitment to early endosomes.

## Discussion

It is well-known that intestinal microbiota releases many regulatory signals that modulate the development and function of the intestinal immune system. To house this huge microbial population, the intestinal tract needs barrier and regulatory mechanisms to control reciprocal interactions between microbiota, the epithelium and the mucosal immune system, preventing aberrant responses and maintaining homeostasis. In the gut, host–microbiota crosstalk mainly depends on secreted factors that can diffuse across the mucus layer and gain access to the epithelium. Among bacterial secreted factors, bacterial extracellular vesicles have relevant role in bacteria–host communication, as they allow the intracellular delivery of bacterial effector upon internalization into the host cells. OMVs released by Gram-negative bacteria enclose many ligands recognized by PRRs such as LPS (TLR4 ligand), PG (NOD1/NOD2 ligand), or DNA (TLR9 ligand). These receptors, expressed by epithelial and immune cells, are crucial components of innate immunity as they detect gut microbes and elicit suitable immune responses.

NOD1 and NOD2 cytosolic receptors detect PG, a component of the cell wall of Gram-negative and Gram-positive bacteria (Girardin et al., 2003). Many studies have shown the pivotal role of these immune receptors in defense against bacterial infections and in the modulation of host inflammatory responses (Caruso et al., 2014; Philpott et al., 2014). In addition to this function as sensors of bacterial pathogens, recent contributions evidenced that NODs are also fundamental to maintain intestinal homeostasis and microbiota balance (Rehman et al., 2011; Philpott et al., 2014). In fact, mutations that affect NOD2 expression or activity have been associated with multiple chronic inflammatory and autoimmune diseases (Feerick and McKernan, 2016). In this context, boosting the stimulation of NOD receptors by microbiota has been proposed as a mechanism to ensure gut homeostasis by enhancing innate immunity (Clarke et al., 2010). Nevertheless, how gut microbiota, which is composed of non-invasive bacteria, can deliver PG into host cells is a rather unexplored issue. A plausible pathway is that PG fragments released into the intestinal lumen during bacterial cell division are internalized by epithelial cells through endocytosis or by oligopeptide transporters such as PEPT1 or PEPT2 (Swaan et al., 2008; Philpott et al., 2014). NOD-activating PG products have been found in *E. coli* culture supernatants (Pradipta et al., 2010), and PG fragments released by *L. salivarius* protect colitis progression in mice by upregulating IL-10 (Macho-Fernández et al., 2011).

Another pathway for intracellular PG delivery is through bacterial vesicles. This a well-studied mechanism in Gram-negative pathogens such as *H. pylori*, *V. cholerae*, and *A. actinomycetemcomitans* (Kaparakis-Liaskos et al., 2010; Bielig et al., 2011; Thay et al., 2014). Studies performed with *H. pylori* revealed that internalized OMVs reach endosomal compartments, and that interaction with OMV-PG takes place at early endosomes (Irving et al., 2014). With this information, we hypothesized that microbiota-released OMVs could mediate immunomodulatory effects through NOD activation by their PG. Previous contributions of our group showed that OMVs from commensal and probiotic *E. coli* strains enter the intestinal epithelial cells via clathrin-dependent endocytosis (Cañas et al., 2016) and elicit immunomodulatory and barrier reinforcement responses (Alvarez et al., 2016; Fábrega et al., 2016). Several vesicle cargo molecules and MAMPs can account for these effects, thus involving a number of immune receptors and signaling pathways. To evaluate involvement of the NOD signaling pathways in the immune response triggered by commensal *E. coli* strains we used the epithelial cell line Caco-2, which poorly responds to LPS due to low expression of TLR4 and their co-receptor MD-2 (Abreu et al., 2001; Kim et al., 2004).

Our results show that OMVs from the commensal ECOR12 and the probiotic EcN induce an innate immune response in Caco-2 cells by activating NOD1-signaling cascades, which led to activation of NF-κB and secretion of pro-inflammatory cytokines IL-6 and IL-8. Several factors led us to rule out the involvement of NOD2 in these responses: (i) intestinal epithelial cells express very low levels of NOD2, (ii) NOD2 expression is not upregulated by microbiota *E. coli* OMVs, and (iii) NOD2 silencing did not affect OMV-activated IL-6 and IL-8 responses. In contrast, NOD1 silencing and RIP2 inhibition significantly decreased expression of these pro-inflammatory cytokines at both mRNA and protein levels. Interestingly, EcN and ECOR12 OMVs elicited greater activation of IL-8 than the specific NOD1 ligand Tri-DAP (used at 1 μg/ml), thus indicating the relative contribution of MAMPs other than PG to OMV-mediated activation of IL-8 secretion. Activation by OMVs of other signaling pathways that lead to IL-8 expression may explain why IL-8 secreted levels were not significantly reduced by treatments that interfere with NOD1 levels (NOD1-kockdown cells) or RIP2 activity (Gefitinib-treated cells).

In cells stimulated with EcN or ECOR12 OMVs, NOD1-mediated activation of NF-κB was confirmed by measuring IκBα degradation. As expected, both OMVs triggered degradation of this IκB inhibitory protein, and the effect was not observed in RIP2 inhibited cells. Interestingly, both OMVs triggered IκBα degradation with different kinetics, which were faster for EcN OMVs. In cells treated with vesicles from the probiotic EcN, a reduction in IκBα levels was apparent at 40 min post-stimulation, with values that were statistically significant at 90 min and later times. However, vesicles from the commensal ECOR12 did not cause any significant changes in IκBα levels until 120 min. At 120 min, IκBα levels were reduced by more than 60% in cells treated with EcN OMVs, but by around 40% in cells treated with ECOR12 OMVs. At 180 min, no differences in IκBα levels were observed between EcN- or ECOR12-treated cells. The different IκBα degradation timing profile was compatible with the kinetics of NOD1 aggregation in OMVs-treated cells. NOD1 aggregation may be used as a sign of NOD1 activation that leads to NOD1 oligomerization and recruitment of other proteins to form large signaling complexes (Sorbara et al., 2013; Irving et al., 2014). Analysis by confocal fluorescence microscopy evidenced that the kinetics of NOD1 aggregation in response to internalized ECOR12 OMVs was slower than that triggered by EcN OMVs. Up to 90 min, the smaller number of NOD1 activated complexes observed in the cytosol of ECOR12-treated cells correlated well with the lower IκBα degradation ratios estimated by Western blot at the same incubation times.

The intracellular compartment where the PG contained in OMVs internalized via clathrin-mediated endocytosis interacts with cytosolic NOD1 was elucidated through an elegant study performed with *H. pylori* OMVs. Results from this study revealed that such interaction takes place at the membrane of early endosomes, where NOD1 and RIP2 are recruited (Irving et al., 2014). Regarding microbiota-derived OMVs, confocal fluorescence microscopy analysis performed at 60 min post-stimulation confirmed that OMVs from both EcN and ECOR12 colocalize with the endosome marker EEA1 and with NOD1. At this incubation time, NOD1 was found associated with early endosomes only in OMVs-treated cells. The overlap coefficients calculated for the colocalization of OMVs with EEA1 match those reported previously at 30 min (Cañas et al., 2016), and are compatible with the transient location of OMVs in early endosomes during their trafficking to lysosomes. Similar overlapping coefficient values were obtained here for EEA1/NOD1 signals, which is consistent with the fact that NOD1 associates to early endosomes to interact with PG contained in OMVs.

It is known that NOD receptors recognize muramyl peptides derived from bacterial PG. These active degradation products can be generated by the action of amidases or lytic enzymes either of bacterial (muramidases) or from mammalian origin (lysozyme type enzymes) (Girardin et al., 2003). The study carried out with *H. pylori* OMVs did not reveal how NOD1-activating peptides are generated from PG-OMVs and how they are presented to NOD1 (Irving et al., 2014). In this sense, both endosomal enzymes and the acidic pH of endosomes (pH 5.5–6) seem to be essential for the processing of NOD1-activating peptides derived from bacterial PG (Lee et al., 2009). In addition, bacterial OMVs contain PG hydrolytic enzymes that can account for the release of muramyl peptides (reviewed in Jan, 2017). Enzymatic generation of these peptides into the endosomal compartment, together with the presence of a transport system for oligopeptides in the endosome membrane (SLC15A4), which acts as a gateway for the release of these active peptides to the cytosol (Lee et al., 2009), may help NOD1 interaction and its activation to initiate the phosphorylation cascade that leads to activation of NF-κB.

Clearly, the formation and processing of NOD1-activating muramyl peptides from internalized OMVs require bacterial and/or endosomal enzymes. Thus, differences in the OMVs cargo or in the envelope vesicular structure may influence the availability of active muramyl peptides. As stated above, vesicles from the probiotic EcN and the commensal ECOR12 elicited similar IL-6 and IL-8 responses, although they differed in their initial kinetics of NOD1 and NF-κB activation. We may speculate that these differences could be attributed to different vesicular cargo, especially in the content of PG-degrading enzymes. OMVs from the probiotic EcN contain several lytic murein transglycosylases such as MtlA, MtlB and EmtA (Aguilera et al., 2014), but no information is available so far on the ECOR12 OMV- proteome. Other factors related with the vesicular envelope may also contribute to the different activating profile. For example, the probiotic EcN contains a K5 polysaccharide capsule that is absent in the commensal ECOR12. The presence of this capsule on the surface of EcN OMVs may influence the action of PG processing enzymes. The faster the active PG-derived peptides are produced the less time is needed for NOD1 activation.

## Conclusion

Modulation of the innate immune system by gut microbiota has essential role in maintaining gut homeostasis, both helping quick responses against pathogens and preserving hyporesponsiveness to commensal bacteria and innocuous antigens. In this study, we provide evidence that OMVs released by beneficial gut bacteria provide a mechanism for PG delivery into the host cytosol, thus allowing the sensing of microbial products that steadily prime the innate immune system. The constant stimulation of NOD1 by microbiota vesicles results in controlled inflammatory responses that may help pathogen eradication and host survival. Therefore, by this mechanism microbiota OMVs contribute to immune responses essential to maintaining intestinal homeostasis.

## Author Contributions

LB and JB conceived of the study with the contribution of RG, M-AC, and M-JF in experimental design. LB and JB wrote the manuscript and supervised the work. RG, M-AC, and M-JF carried out the data interpretation and statistical analysis. M-AC and M-JF executed the experimental work. All authors revised, read, and approved the final manuscript.

## Conflict of Interest Statement

The authors declare that the research was conducted in the absence of any commercial or financial relationships that could be construed as a potential conflict of interest.

## Abbreviations

DMEM: Dulbecco’s Modified Eagle Medium
DSS: dextran sulfate sodium
DTT: dithiothreitol
EcN: *Escherichia coli* Nissle 1917
FBS: fetal bovine serum
LB: Luria-Bertani broth
LPS: lipopolysaccharide
MAMPs: microbial-associated molecular patterns
NLR: NOD-like receptor
NOD: nucleotide oligomerization domain
OMVs: outer membrane vesicles
PAGE: polyacrylamide gel electrophoresis
PBS: phosphate buffer saline
PG: peptidoglycan
PRR: pattern recognition receptor
RIP2: receptor-interacting protein 2
RT-qPCR: quantitative reverse transcription PCR
SDS: sodium dodecyl sulfate
TLR: Toll-like receptor
ZOs: zonula occludens
