# Supplementary material for: Outer Membrane Vesicles From Probiotic and Commensal Escherichia coli Activate NOD1-Mediated Immune Responses in Intestinal Epithelial Cells
Source: Front Microbiol. 2018 Mar 20;9:498. doi: 10.3389/fmicb.2018.00498 (PMC5869251; doi:10.3389/fmicb.2018.00498)
Supplement: Supplementary file 1 [file Image_1.PDF]

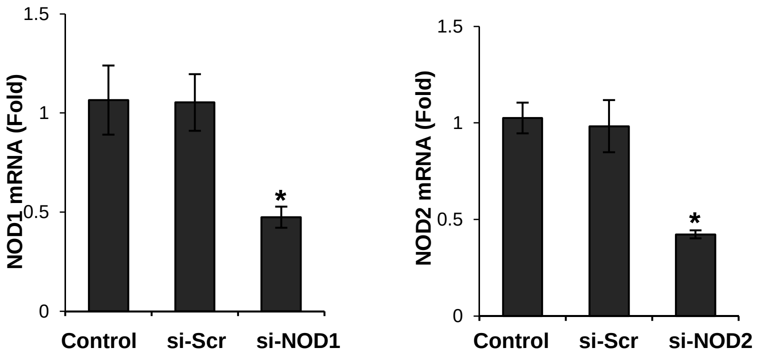

Figure S1. Quantitative PCR of NOD1 and NOD2 mRNAs in Caco-2 cells transfected with siRNA specific sequences targeting these NOD receptors, or with control scrambled siRNA (Scr).
