# Supplementary material for: Outer Membrane Vesicles From Probiotic and Commensal Escherichia coli Activate NOD1-Mediated Immune Responses in Intestinal Epithelial Cells
Source: Front Microbiol. 2018 Mar 20;9:498. doi: 10.3389/fmicb.2018.00498 (PMC5869251; doi:10.3389/fmicb.2018.00498)
Supplement: Supplementary file 2 [file Image_2.PDF]

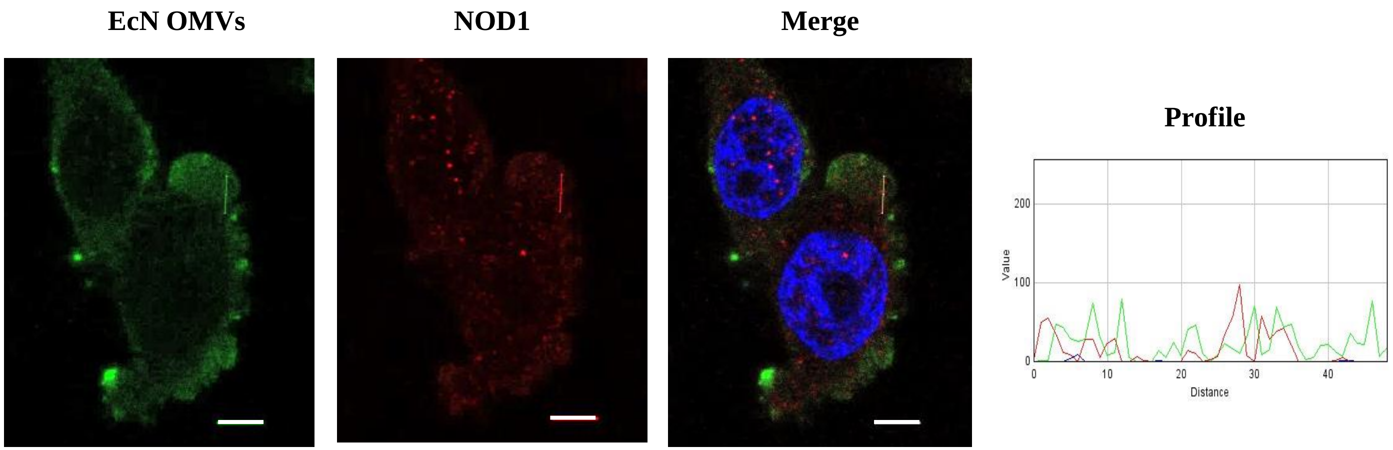

Figure S2. Colocalization of EcN OMVs with NOD1. HT-29 cells were incubated with BODIPY®FL-labelled OMVs for 1h and analyzed using laser scanning confocal spectral microscope. NOD1 was stained using anti-NOD1 anti-NOD1 policlonal antibody and AlexaFluor 633-conjugated goat anti-rabbit IgG (red) and nuclei with DAPI (blue). Images are from a single representative experiment (n=3). Colocalization of the green (PG-OMVs) and red (NOD1) signals was confirmed by histogram analysis of the fluorescence intensities along the yellow line. Analysis was performed by laser scanning confocal spectral microscope with 63x oil immersion objective lens, and images were captured with a Nikon color camera (8 bit). Scale bar: 10 µm.
